# Validation of the Japanese Version of the Low Anterior Resection Syndrome Score

**DOI:** 10.1007/s00268-018-4519-8

**Published:** 2018-02-14

**Authors:** Emi Akizuki, Hiroshi Matsuno, Tetsuta Satoyoshi, Masayuki Ishii, Akihiro Usui, Tomomi Ueki, Toshihiko Nishidate, Kenji Okita, Tsunekazu Mizushima, Masaki Mori, Ichiro Takemasa

**Affiliations:** 1 Department of Surgery, Surgical Oncology and Science, Sapporo Medical University, Sapporo, Japan; 2 Department of Gastroenterological Surgery, Osaka University Graduate School of Medicine, Osaka, Japan

## Abstract

**Background:**

The low anterior resection syndrome (LARS) score is a patient-reported outcome measure to evaluate the severity of bowel dysfunction after rectal cancer surgery by scoring the major symptoms of LARS. The aim of this study was to translate the English version of the LARS score into Japanese and to investigate the validity and reliability of the LARS score.

**Methods:**

The LARS score was translated in Japanese following current international recommendations. A total of 149 rectal cancer patients completed the LARS score questionnaire and were also asked a single question assessing the impact of bowel function on quality of life (QoL). A total of 136 patients answered the LARS score questionnaire twice.

**Results:**

The Japanese LARS score showed high convergent validity, based on its good correlation between the LARS score and QoL (*p* < 0.001). The LARS score was able to discriminate between patients according to the tumor distance to anal verge (*p* < 0.001), type of surgery (*p* < 0.001), and time since surgery (*p* = 0.001). Patients after ultra-low anterior resection and intersphincteric resection showed especially high scores. The score also had high test–retest reliability (intraclass correlation coefficient: 0.87).

**Conclusion:**

The Japanese LARS score is a valid and reliable tool for measuring LARS. The LARS score is appropriate for assessments in postoperative bowel function and international comparison. Using this score, patient-reported outcome measures of LARS in Japanese patients can be shared internationally. Additional validation reports from non-English speaking countries can support the LARS score as a worldwide assessment tool for postoperative bowel dysfunction.

## Introduction

Colorectal cancer is the most common cancer in Japan, and approximately 44,000 cases of cancer located in rectosigmoid and rectum are diagnosed annually [1, 2]. Up to 80% of rectal cancer patients currently undergo sphincter-preserving surgery, which includes anterior resection (AR); low anterior resection (LAR); and for very low rectal cancer, ultra-low anterior resection (ULAR) and intersphincteric resection (ISR). These surgeries were developed due to a better understanding of cancer biology, improved surgical technology, and the patient’s desire to avoid a permanent stoma and have better quality of life (QoL).

Up to 90% of patients after sphincter-preserving surgery have changes in bowel habits. Symptoms range widely from increased bowel frequency to fecal incontinence or evacuatory dysfunction and urge. The combination of symptoms after sphincter-preserving surgery is referred to as low anterior resection syndrome (LARS). Previously, LARS was thought to be transient, and most patients resolve within 1 year. However, recent long-term studies show that adverse symptoms continue, and LARS is not a short-lived neorectal irritability, but a result of permanent changes in the postoperative period [3].

Although LARS may severely affect a patient’s QoL, a reliable estimate of prevalence and patient impact does not exist. One of the major reasons for this is the fact that there is no broadly accepted outcome measure for postoperative bowel dysfunction [4, 5]. A number of instruments were applied to measure functional bowel outcomes in the past reports; the Cleveland Clinic Florida Fecal Incontinence Score (Wexner incontinence score) [6], the St Marks’ Fecal Incontinence Grading Score [7], the Rockwood Fecal Incontinence Severity Index [8], or the Fecal Incontinence Quality of Life Scale (FIQL) [9]. However, these scores were originally produced as a measure of simple incontinence, and they are too narrow and specific for assessing complicated dysfunctions such as LARS.

On 2012, Emmertsen et al. [10] developed “the LARS score” in Danish. The LARS score is a patient-reported outcome measure to evaluate the severity of bowel dysfunction after rectal surgery by scoring the major symptoms of LARS: incontinence (flatus and liquid stool), frequent bowel movements, fragmentation/clustering of the stools, and urge. The original Danish version showed was translated into English, and the English version has been translated in 16 languages, and 7 languages (Danish [10], English [11], Swedish, Spanish, German [12], Chinese [13], and Lithuanian [14]) are formally validated. Validation of multi-languages will enable internationally standardized reports of LARS irrespective of the native language. Moreover, reports from various regions in the world will support the understanding of LARS and can assist the LARS score as a well-validated international assessment tool.

The aim of this study was to translate the English version of the LARS score into Japanese and investigate its validity in rectal cancer patients in Japan.

## Methods

### Translation

We granted permission by the original LARS score authors to translate the LARS score into Japanese. The English version of the LARS score (Table 1) [10] was translated to Japanese by two independent professional translators whose native language was Japanese. The translators discussed any discrepancies between the two versions. A common version was then established and was back-translated to English by a third independent translator whose native language was English. The third translator was not familiar with the English version. The back-translations were done to check whether the original meaning of each question was preserved. The translations followed the recommendations of the WHO and the European Organization for Research and Treatment of Cancer (EORTC) [15–17].

**Table 1:** English version of the LARS score

The aim of this questionnaire is to assess your bowel function. Please tick only one box for each question. It may be difficult to select only one answer, as we know that for some patients symptoms vary from day to day. We would kindly ask you to choose one answer which best describes your daily life. If you have recently had an infection affecting your bowel function, please do not take this into account and focus on answering questions to reflect your usual daily bowel function	
Q.1: Do you have occasions when you cannot control your flatus (wind)?	
□ No, never	0
□ Yes, less than once per week	4
□ Yes, at least once per week	7
Q.2: Do you ever have any accidental leakage of liquid stool?	
□ No, never	0
□ Yes, less than once per week	3
□ Yes, at least once per week	3
Q.3: How often do you open your bowels?	
□ More than 7 times per day (24 h)	4
□ 4-7 times per day (24 h)	2
□ 1–3 times per day (24 h)	0
□ Less than once per day (24 h)	5
Q.4: Do you ever have to open your bowels again within 1 h of the last bowel opening?	
□ No, never	0
□ Yes, less than once per week	9
□ Yes, at least once per week	11
Q.5: Do you ever have such a strong urge to open your bowels that you have to rush to the toilet?	
□ No, never	0
□ Yes, less than once per week	11
□ Yes, at least once per week	16

Add the scores from each of the five answers to one final score

Interpretation: 0–20 = No LARS 21–29 = Minor LARS 30–42 = Major LARS

The final versions were checked and accepted by the corresponding author (Japanese version of the LARS score, http://sapmed-surg1.jp/medical/lars.shtml).

### Participants

Patients with rectal cancer within 15 cm from the anal verge following with curative sphincter-preserving surgery were included in the study. Exclusion criteria were disseminated or recurrent disease, presence of stoma less than 1 year after surgery or stoma closure, aged <20 years, and patients with mental dementia or inability to read/speak/understand the Japanese language. Participants were identified through the medical records of rectal cancer patients who were hospitalized at the gastrointestinal surgery department of Osaka University. Demographic and clinical information was obtained from the database. During the period from January 1, 2010, to December 31, 2013, 321 patients were treated surgically and were included in this study on 2015.

Participants were contacted by posted mail to inform them about the purpose of this study and asked to fill out the questionnaire. The questionnaire was returned by mail. Nonresponders were further contacted by phone. Data collection and the contacting of participants were conducted by an independent data center.

### Questionnaire

All participants were sent an invitation to participate in the study, and the Japanese LARS score questionnaire was enclosed (Japanese version of the LARS score, http://sapmed-surg1.jp/medical/lars.shtml). In addition, a separate question to assess their QoL (“Overall, how much does your bowel function affect your quality of life?”) was included. The available responses were “not at all,” “a little,” “some,” “a lot.” This extra question was added for validation purposes to enable the investigation of the association between the LARS score and QoL.

### Test–retest

To examine the test–retest reliability of the score, all participants who returned the first test were mailed the LARS score questionnaire again. The second test was mailed to the participants 1–2 weeks after the completion of the first test. If the time interval between the completions of the two tests was outside the predefined interval of 1–8 weeks, data were excluded from the analysis.

### Statistical analysis

#### Convergent validity

The LARS score was computed and categorized into three groups: no LARS (0–20 points), minor LARS (21–29 points), or major LARS (30–42 points), according to the guidelines [10]. To facilitate the analysis of convergent validity, participants were further categorized into three QoL groups: categorization 1 “no, minor, some/major,” or categorization 2 “no, minor/some, major” impact on QoL groups.

For each QoL groups, the LARS score was calculated, and the numerical values of the LARS score were statistically tested. The association between the LARS groups and the QoL groups was investigated by a 3-by-3 table, and the percentage of perfect fit, moderate fit, and no fit was calculated. When the LARS group and the QoL group was in the same categorical level (e.g., “major LARS” and “some/major impact on QoL,” or “major LARS” and “major impact on QoL”) it was regarded as perfect fit. A mismatch in one categorical level was regarded as moderate fit, and more than two levels of mismatch were regarded as no fit.

The sensitivity and specificity of the major LARS for predicting the patients with “some/major impact on QoL,” or “major impact on QoL” were also assessed by receiver operating characteristic (ROC) curves.

#### Discriminative validity

Discriminative validity was evaluated by comparing groups which were expected to differ with regards to LARS: sex, age (over or less than 70 years), tumor distance from the anal verge (higher or lower than 8 cm), type of surgery, and time since surgery (time since stoma-free rectal resection surgery or reversal surgery of temporary stoma, early or late than 2.5 years). The definition for each surgery is as following, AR: partial proctectomy with anastomosis above the peritoneal reflection, LAR: partial proctectomy with anastomosis below the peritoneal reflection [18], ULAR: complete proctectomy with the colon directly connected to the anal canal with the anastomosis 2 cm proximal to the dentate line [19], ISR: complete proctectomy with a partial or total resection of the internal anal sphincter using a perineal approach, and the anastomosis was at the same level of the dentate line or even distal with a hand-sewn coloanal anastomosis [20, 21]).

#### Test–retest reliability

In the analysis of the test–retest reliability, the extent of agreement between the LARS score at the first and second test was demonstrated on a Bland–Altman plot with limits of agreement. The correlation between the numerical values of the first and second LARS scores was assessed by the intraclass correlation coefficient. The difference between the numerical values of the LARS scores for the two tests was tested by the paired t test.

In addition, the agreement between the first and second response was explored by computing the percentages of perfect, moderate, and no agreement. When the answer was same for both the first and second test it was judged as perfect agreement, a difference for one category was moderate agreement, and difference over two categories was no agreement.

Differences were tested using the Mann–Whitney *U* test or Kruskal–Wallis test, depending on the data type and distribution. All *p* values <0.05 were considered statistically significant. All statistical analyses were performed using IBM SPSS Statistics 18 (SPSS Japan Institute, Tokyo, Japan,).

### Ethics

This study was approved by the biomedical ethics committee of the Osaka University (IRB15074). Informed consent was obtained from all patients who participated in the study at the time of the first contact by mail.

## Results

### Translation

The double-forward translations revealed no discrepancies. The backward translations to English were an exact match and confirmed that the original meaning of each of the five questions was retained.

### Participants

There were 321 consecutive rectal cancer patients, and 196 patients met the criteria and were eligible for the study. Of the 196 eligible patients, 153 responded (78.1%). Four returned incomplete questionnaires, and 149 patients were included in the statistical analysis (76.0%). The clinical and demographic characteristics of the participants are presented in Table 2. According to the LARS score, 55 (36.9%) patients had “no LARS,” 36 (24.2%) had “minor LARS,” and 58 (38.9%) had “major LARS.”

**Table 2:** Patient and treatment characteristics of participants (*n* = 149)

	Values
Male/female [*n*(%)]	94/55 (67/33)
Age at surgery [years (range)]	64 (28–90)
Tumor stage [*n*(%)]	
*T*0–*T*2	90 (60)
*T*3–*T*4	59 (40)
Tumor distance to anal verge [cm (range)]	10 (3–15)
Type of surgery [*n*(%)]	
AR	39 (26)
LAR	67 (45)
ULAR	33 (22)
ISR	10 (7)
Time since surgery [years (range)]	3.8 (1.3–6.9)

### Convergent validity

**Fig. 1:**
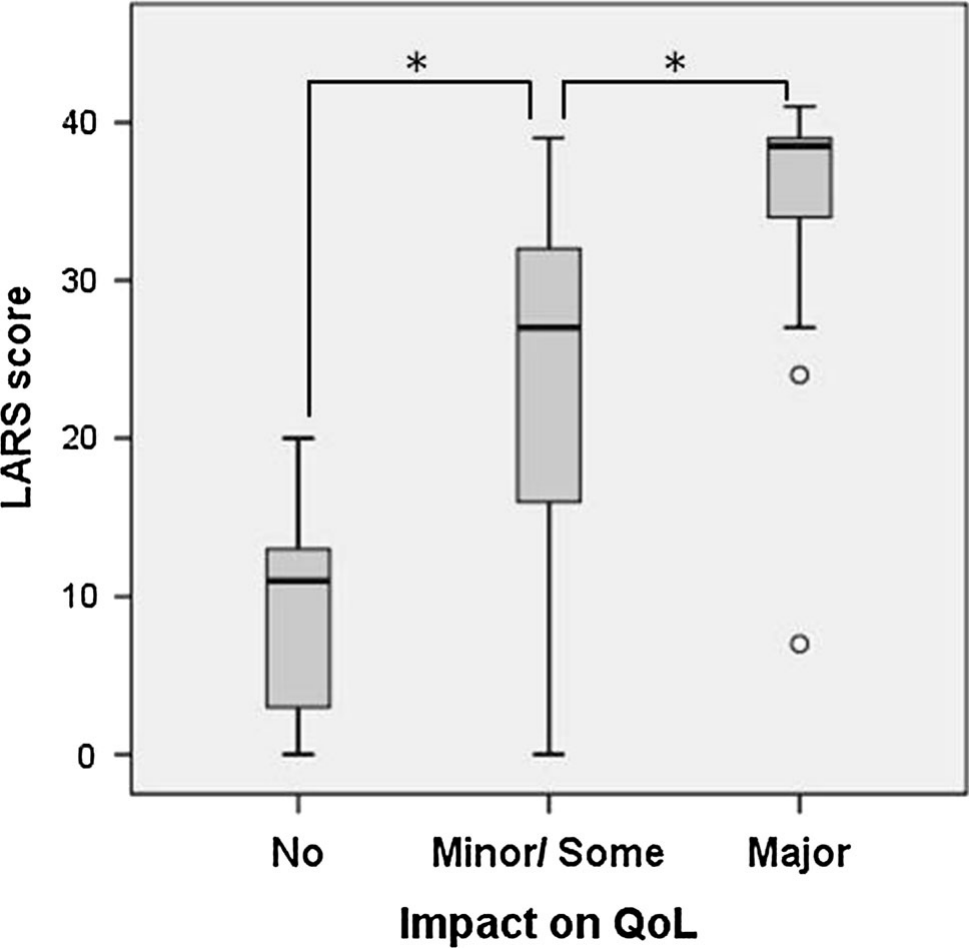
LARS score versus impact in QoL. There was a significant difference between each groups (*p < 0.001, Mann–Whitney *U* test)

The median LARS scores for each QoL groups are as following; No impact on QoL (*n* = 20): median LARS score 11 (range 0–20), minor impact on QoL (*n* = 34): 19 (0–37), Some impact on QoL (*n* = 65): 29 (5–39), major impact on QoL (*n* = 30): 38 (7–41). With the QoL categorization 1, the LARS scores were, No: 11 (0–20), minor: 19 (0–37), some/major: 31 (5–41), and the scores between each category were statistically significant (no vs. minor *p* = 0.02, minor vs. some/major *p* < 0.001, Mann–Whitney *U* test). With the QoL categorization 2, the LARS scores were, No: 11 (0–20), minor/some: 27 (0–39), major: 38 (27–41), and the results are shown in Fig. 1.

With the QoL categorization 1, the fit between the LARS group and QoL group was 55.7% perfect fit, 32.2% moderate fit, and 12.1% no fit. With the categorization 2, the results are shown in Table 3.

**Table 3:** Agreement between the QoL group and the LARS category

	Impact of bowel function on QoL		
	No	Minor/some	Major
No LARS	20 (13.4%)	34 (22.8%)	1 (0.7%)
Minor LARS	0 (0%)	33 (22.1%)	3 (2.0%)
Major LARS	0 (0%)	32 (21.5%)	26 (17.4%)

Perfect fit: 53.0%; moderate fit: 46.3%; no fit: 0.7%

**Fig. 2:**
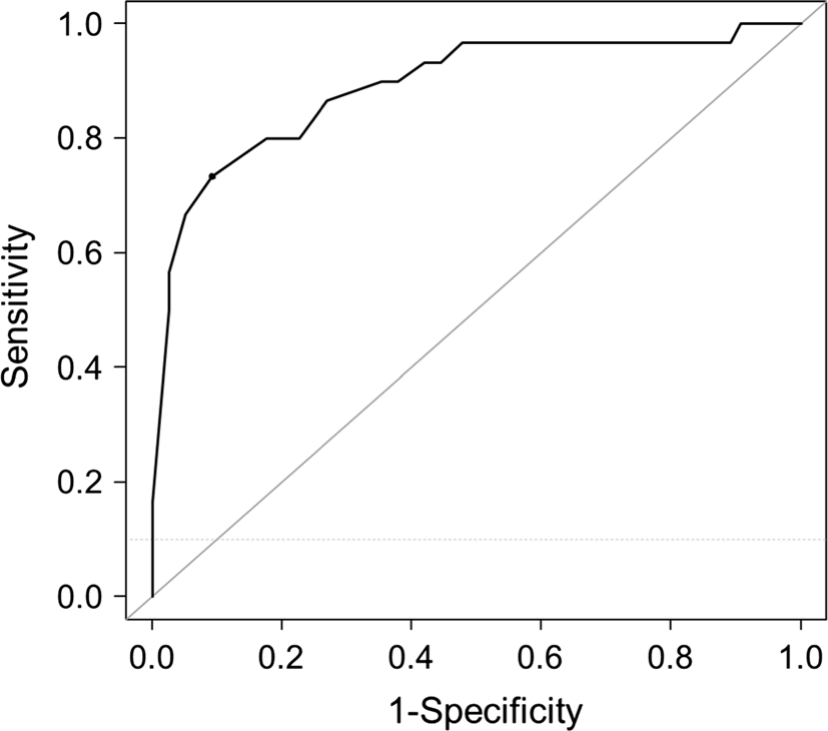
ROC curve shows the relation between LARS score and major impact on QoL. Area under the curve = 0.891

The ROC curve of LARS score predicting patients with “some/major impact on QoL” showed an area under curve AUC 0.83 (95% CI 0.77–0.90), with 54.7% sensitivity and 88.8% specificity. For predicting patients with “major impacts on QoL,” the ROC curve is shown in Fig. 2, with 86.6% sensitivity and 73.1% specificity.

### Discriminative validity

**Fig. 3:**
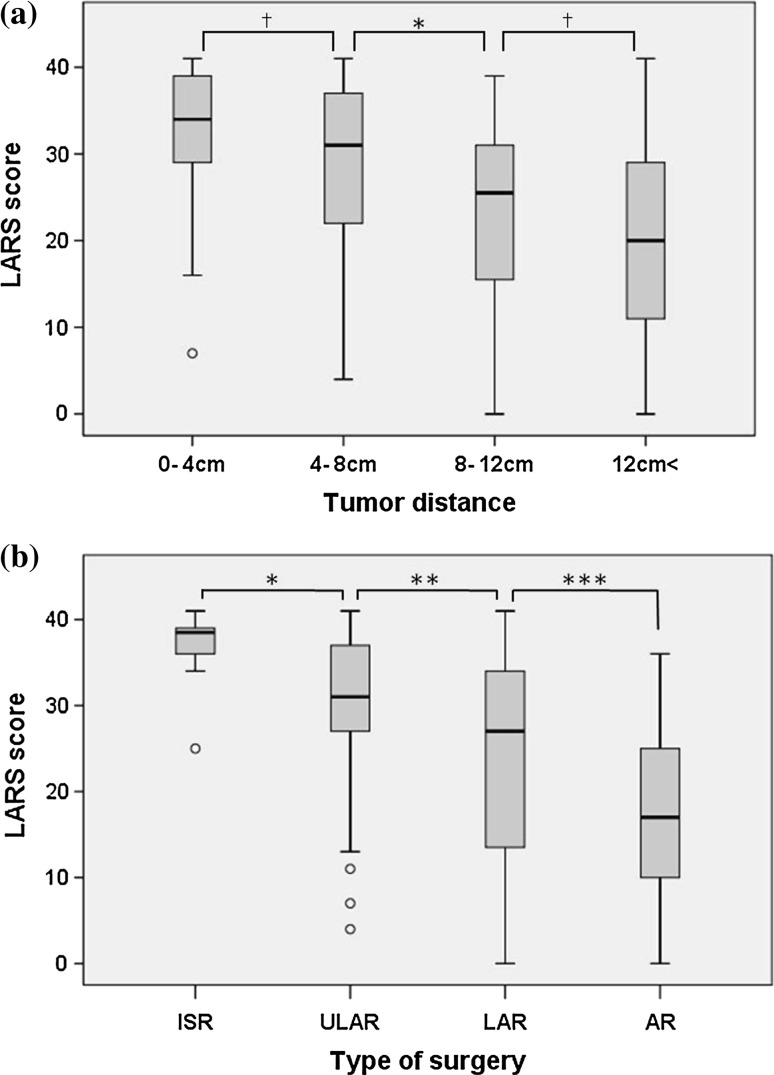
Comparison of LARS score in **a** tumor distance to anal verge (*p = 0.018, ^†^*p* > 0.05*)*, and** b** type of surgery (*p = 0.008, **p = 0.045, ***p = 0.004) (Mann–Whitney *U* test)

Patients grouped according to the tumor distance to anal verge, type of surgery, and time since surgery showed significant differences in LARS scores (Table 4). The LARS score gradually increased depending on the tumor distance from anal verge (Fig. 3a), and the type of surgery (Fig. 3b).

**Table 4:** Discriminative validity of the LARS score

Group		LARS score	
	*n* (%)	Median (range)	*P*
Sex			0.08*
Male	94 (63)	29 (0–41)	
Female	55 (37)	23 (0–39)	
Age (years)			0.34*
<70	111 (74)	27 (0–41)	
>70	38 (26)	24 (0–41)	
Tumor stage			0.11*
*T*0–*T*2	96 (64)	26 (0–41)	
*T*3–*T*4	53 (36)	29 (0–41)	
Tumor level (cm)			<0.001*
<8	61 (41)	32 (4–41)	
>8	88 (59)	22 (0–41)	
Type of surgery			<0.001**
ISR	10 (7)	38 (25–41)	
ULAR	33 (22)	31 (4–41)	
LAR	67 (45)	27 (0–41)	
AR	39 (26)	17 (0–36)	
Time since surgery (years)			0.001*
<2.5	39 (26)	32 (7–41)	
>2.5	110 (74)	23 (0–41)	

*Mann–Whitney *U* test

**Kruskal–Wallis test

### Test–retest reliability

**Fig. 4:**
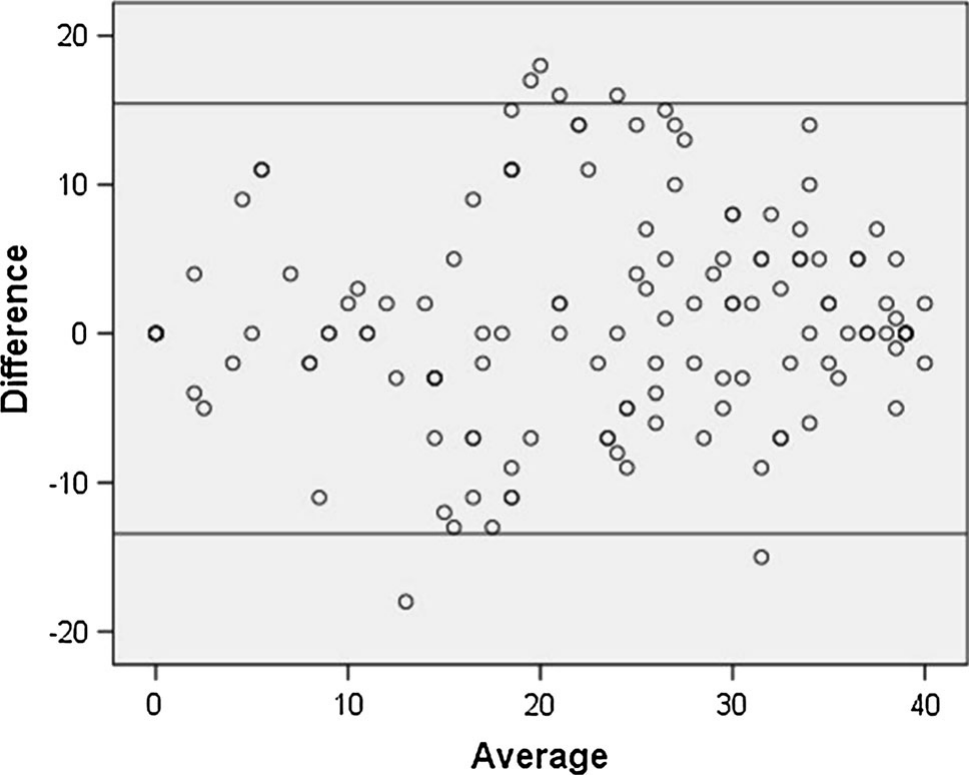
Bland Altman plot with 95% limits of agreement (− 13.4 to 15.4) illustrating the difference between the LARS score at the first and second test

A total of 149 patients were asked to complete the LARS score twice, and 136 responded to both questionnaires (response rate 91.3%). Figure 4 illustrates the Bland–Altman plot of the differences between LARS scores on the first and second tests. There was no statistically significant difference between LARS score on the first and second test (*p* = 0.11, paired *t* test). The intraclass correlation was 0.87 (95% CI 0.81–0.91), indicating excellent reliability. The degree of agreement between the initial test and the retest for each of the five LARS score items, and the LARS groups are presented in Table 5.

**Table 5:** Degree of agreement between the response of the first and second test

	Agreement		
	Perfect (%)	Moderate (%)	No (%)
Q.1	69.1	18.4	12.5
Q.2	71.3	22.1	6.6
Q.3	72.1	27.2	0.7
Q.4	72.8	22.8	4.4
Q.5	69.1	30.1	0.7
LARS category	63.2	33.1	3.7

## Discussion

This study translated the English version of the LARS score into Japanese and validated the Japanese version of the LARS score for rectal cancer patients in Japan. The five items of the LARS score consisted of simple and straightforward phrases; therefore, the translation into Japanese was easily carried out.

The results of this study are very similar to those presented in previous publications. We believe that the Japanese LARS score is semantically equivalent to the English and other versions, and a practical international tool. The high response rate and completion rate demonstrate that the Japanese LARS score is easy to understand and to complete, and feasible to use in daily clinical practice for identifying patients with LARS.

In this study, we analyzed two types of QoL categorization. The QoL measure in this study is not a validated questionnaire, and in our understandings, “some” is an intermediate value which has a potential for both “minor” and “major.” We believe there is no discrepancy in categorizing the “some” population in either groups, and in our study, categorizing “no, minor/some, major” was appropriate to focus on patients with major LARS. However, this is the limitation of our study that we used an unvalidated measure to assess QoL. Several reports comparing LARS score and validated QoL measures such as the EORTC QLQ-C30 already exist [12, 22, 23]. An additional analysis with a validated QoL score such as the Medical Outcomes Study Short Form 36 (MOS SF36) [24, 25], or with other fecal incontinence, such as FIQL [26–28] can provide novel information for the validity of the LARS score.

In previous studies, a high LARS score was associated with radiotherapy, tumor height, total mesenteric excision (TME), and elder age [10–12]. However, the concept of TME/partial mesenteric excision (PME) is relatively new in Japan, and information was not available in our database. Our type of surgery classifications was AR/LAR/ULAR/ISR, which was based on the type of resection. Differences in surgical procedures should affect the functional outcomes, and as expected, the Japanese LARS score gradually increased depending on the type of surgery. These results suggest that the LARS score is able to discriminate between patients undergoing different types of surgery. The ULAR and ISR procedures showed especially high LARS scores; moreover, patients undergoing ISR had significantly higher LARS scores than those undergoing ULAR. The difference between ISR and ULAR is the resection of the internal anal sphincter, and the hand-sewn anastomosis [20, 21]. Both factors may affect the postoperative bowel function, and further estimation in ISR is expected.

Unfortunately, our study was a single center study, and our cases included few neoadjuvant chemotherapy nor neoadjuvant radiotherapy. Further data collection is necessary to assess the population of major LARS in Japan, and the risk factors including radiotherapy [29], chemotherapy, diverting stoma [30], and anastomotic leakage [31].

## Conclusion

The Japanese version of the LARS score was proven to be a valid and reliable for measuring LARS in Japanese rectal cancer patients. The LARS score can be used in daily clinical practice, and scientific studies to identify and follow-up patients with LARS. From now on, patient-reported outcome measures of LARS in Japan can be shared internationally, and additional translation and validation report of a new language can support the LARS score as a worldwide assessment tool for postoperative bowel dysfunction.
